# “APEC Blue”: Secondary Aerosol Reductions from Emission Controls in Beijing

**DOI:** 10.1038/srep20668

**Published:** 2016-02-18

**Authors:** Yele Sun, Zifa Wang, Oliver Wild, Weiqi Xu, Chen Chen, Pingqing Fu, Wei Du, Libo Zhou, Qi Zhang, Tingting Han, Qingqing Wang, Xiaole Pan, Haitao Zheng, Jie Li, Xiaofeng Guo, Jianguo Liu, Douglas R. Worsnop

**Affiliations:** 1 State Key Laboratory of Atmospheric Boundary Layer Physics and Atmospheric Chemistry, Institute of Atmospheric Physics, Chinese Academy of Sciences, Beijing 100029, China; 2 Lancaster Environment Centre, Lancaster University, Lancaster LA1 4YQ, UK; 3 College of Applied Meteorology, Nanjing University of Information Science and Technology, Nanjing 210044, China; 4 Department of Environmental Toxicology, University of California, 1 Shields Ave., Davis, CA 95616, USA; 5 Research Institute for Applied Mechanics, Kyushu University, Fukuoka 819-0395, Japan; 6 Anhui Institute of Optics and Fine Mechanics, Chinese Academy of Sciences, Hefei 230031, China; 7 Aerodyne Research, Inc., Billerica, Massachusetts 01821, USA

## Abstract

China implemented strict emission control measures in Beijing and surrounding regions to ensure good air quality during the 2014 Asia-Pacific Economic Cooperation (APEC) summit. We conducted synchronous aerosol particle measurements with two aerosol mass spectrometers at different heights on a meteorological tower in urban Beijing to investigate the variations in particulate composition, sources and size distributions in response to emission controls. Our results show consistently large reductions in secondary inorganic aerosol (SIA) of 61–67% and 51–57%, and in secondary organic aerosol (SOA) of 55% and 37%, at 260 m and ground level, respectively, during the APEC summit. These changes were mainly caused by large reductions in accumulation mode particles and by suppression of the growth of SIA and SOA by a factor of 2–3, which led to blue sky days during APEC commonly referred to as “APEC Blue”. We propose a conceptual framework for the evolution of primary and secondary species and highlight the importance of regional atmospheric transport in the formation of severe pollution episodes in Beijing. Our results indicate that reducing the precursors of secondary aerosol over regional scales is crucial and effective in suppressing the formation of secondary particulates and mitigating PM pollution.

Fine particles (particulate matter with aerodynamic diameter less than 2.5 micrometers) exert large impacts on visibility reduction^1^, radiative forcing^2^, and public health^3^,^4^. High concentrations of PM_2.5_ due to substantial increases in anthropogenic emissions have led to frequent severe haze episodes in China^5^,^6^,^7^,^8^. Aerosol species are often observed to show dramatic increases from tens to hundreds of microgram per cubic meter in 2–3 hours^9^. Such rapid formation and evolution of haze has been observed on a routine cycle in Beijing^6^. Recent studies have noted the dominance of secondary inorganic aerosol (SIA) and secondary organic aerosol (SOA) in PM^9^,^10^,^11^, highlighting the significance of secondary aerosol in haze formation. These findings together indicate the importance of reducing the precursors of secondary aerosol, for example SO_2_, NO_x_, and volatile organic compounds (VOCs), for reducing PM levels and achieving the goals of the national “Atmospheric Pollution Prevention and Control Action Plan”^12^. However, the true contribution of emission controls to PM reduction remains poorly constrained, largely due to the variation of meteorological factors and regional atmospheric transport.

The Beijing 2008 Olympic Games provided the first experimental opportunity to study the impact of emission controls on air pollution. While primary gaseous species and particulates from vehicle emissions and coal combustion were reduced significantly, secondary aerosol actually increased during the first two weeks of full controls^13^,^14^. One reason is that emission controls were mainly restricted to the city of Beijing and had limited impact on secondary aerosol that is formed over regional scales. This is consistent with a large contribution from regional transport to PM_2.5_ in Beijing during haze episodes^9^,^15^. Meteorological conditions were found to play a more important role than emission controls in reducing PM levels during the Olympic Games^16^. These results highlight the large uncertainties in assessing the impact of emission controls on PM reduction over a small region. Compared to the Olympic Games, the Chinese government imposed far stricter emission controls during the 2014 Asia-Pacific Economic Cooperation (APEC) summit in Beijing and surrounding regions^17^,^18^. A series of measures, for example shutting down or halting production from factories and power plants with high emissions, stopping all construction activities, and restricting the number of vehicles on the roads were implemented gradually from 3 November 2014, with the highest level of emission controls for all cities from 6 November. However, how these emission controls affect the chemical composition, sources and formation mechanisms of fine particles under variable meteorological conditions remains poorly understood.

Here we report the deployment of an Aerodyne Aerosol Chemical Speciation Monitor (ACSM) on the Beijing 325 m meteorological tower at 260 m and a High Resolution Aerosol Mass Spectrometer (abbreviated as HR-AMS hereafter) at ground level at the same location (39°58ʹ28″ N, 116°22′16″ E) in urban Beijing from 15 October to 13 November 2014 to measure submicron aerosol composition and evolution simultaneously at two different altitudes. Our data clearly show five pollution episodes briefly separated by clean days through the study period (Fig. 1). Air flows were predominately southerly and meteorological conditions were generally stagnant (low wind speed and high relative humidity) during these episodes. Because emission controls were mainly implemented in cities to the south of Beijing, we use these five episodes to study the impact of regional emission controls on aerosol chemistry in megacity Beijing. This approach isolates the influences of clean periods with air masses from the north and northwest where far fewer emission controls were implemented.

## Results and Discussion

### Aerosol Particle Composition

**Figure 1:**
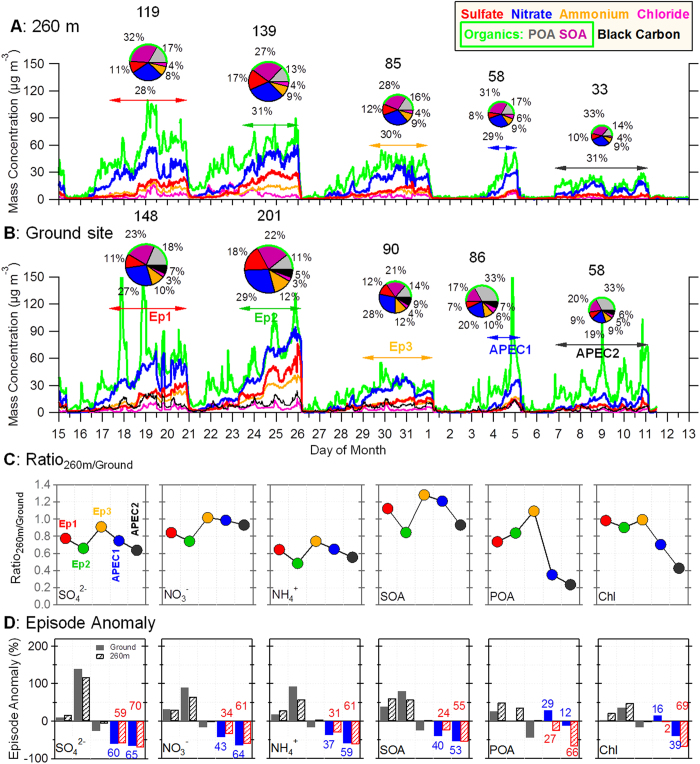
Aerosol particle composition before and during APEC. Time series of aerosol species, including SOA, POA, sulfate, nitrate, ammonium, chloride, and black carbon in PM_1_ at (**A**) 260 m and (**B**) the ground site. The pie charts present the average chemical composition of PM_1_ for five episodes as marked in the Figure, and the numbers on the top of pie chart are the average total PM_1_ mass concentration in microgram per cubic meters. (**C**) shows the concentration ratio of each aerosol species between 260 m and the ground site during each episode. (**D**) shows the average difference in aerosol concentration for each episode compared to the average of all five episodes at 260 m and the ground site. The numbers show the percent change in each chemical species for the two episodes during the APEC.

Submicron aerosol (PM_1_) composition during the three episodes before APEC was dominated by secondary nitrate, which accounted for 27–29% of the PM_1_ mass at the ground site and 28–31% at 260 m (Fig. 1). The nitrate contribution was even higher than the 21–24% found for three pollution episodes during the same period in 2012 (Fig. S9), and that observed at a range of urban and rural sites in China in recent years^19^. Secondary sulfate on average contributed 11–18% to the total PM_1_ mass, about half that of nitrate, but close to the 12–16% observed at the same site in 2012. The increase in NO_3_^−^/SO_4_^2−^ ratios (1.6–2.4 in this study) in recent years demonstrates a clear response of aerosol particle composition to the variations of precursors NO_x_ and SO_2_ emissions during the last decade^20^. Owing to ongoing desulfurization efforts accompanied by increasing NO_x_ emissions from vehicles and industry^21^, nitrate is expected to play a more important role in PM pollution in the near future. Controlling NO_x_ emissions would therefore greatly help mitigate air pollution in China. SOA was the second largest component of PM_1_ accounting for 21–23% and 27–32% at the ground site and 260 m, respectively. The SIA and SOA together contributed 79–83% and 63–72% to the total submicron mass at the ground site and 260 m, highlighting the importance of secondary aerosol formation during haze episodes^9^,^10^.

The two episodes during APEC show very different aerosol composition at the ground site from the earlier episodes, with a large reduction in SIA from 48–59% to 37% and a corresponding increase in organics (Fig. 1). In particular, the contribution of primary organic aerosol (POA) to PM_1_ mass showed a large increase from 11–18% to 33% while that of SOA changed little. These results suggest that emission controls changed aerosol particle composition at the ground site significantly. However, the aerosol particle composition at 260 m didn’t change in the same way, and was similar to that before APEC, dominated by secondary nitrate (29–31%) and SOA (31–33%). The different variations of POA and SOA at the ground site led to a change of oxidation properties of organic aerosol (OA) during APEC. The average oxygen to carbon ratio (O/C), an indicator for the aging degree of OA^22^, decreased from 0.45 to 0.36 during APEC (Fig. S13) indicating that the bulk OA is less oxidized during APEC.

We demonstrate the effects of emission controls by comparing the aerosol composition during the two APEC episodes with the average of all five episodes (Fig. 1). SIA (SO_4_^2−^, NO_3_^−^, and NH_4_^+^) concentrations show an average decrease of 37–60% and 31–59% at the ground site and 260 m, respectively, during the first APEC episode (APEC1 in Fig. 1D) when the highest level of emission controls was initiated in cities located to the southwest of Beijing along the Taihang Mountains. The ground site in Beijing shows an increase in POA of 29% in contrast to a decrease in SOA of 40%. These results indicate the presence of strong local sources despite the emission controls, for example 15–36% increases of hydrocarbon-like OA (HOA) from heavy-duty vehicle emissions, cooking OA (COA), and biomass burning OA (BBOA) were observed (Fig. S10). This is consistent with the small reductions in precursors including CO, NO, NO_2_, and SO_2_ (2–11%) seen during APEC1. In APEC2, after the highest level of emission controls were extended to Tianjin and other major cities in Hebei and Shandong provinces, SIA and SOA showed very similar reductions at the ground site and 260 m, 53–65% and 55–70%, respectively. Primary HOA and BBOA at the ground site however remained relatively constant and even increased slightly during APEC although COA showed a clear reduction (27%). In contrast, POA and chloride at 260 m showed similar reductions as the secondary species suggesting a dominant contribution from regional transport to primary aerosol species at greater heights in urban areas. Our results indicate that controlling precursors over regional scales is effective in reducing PM levels but has very different impacts on primary and secondary aerosol. While secondary aerosol responds directly to regional emission controls, POA at the ground site is more affected by local emission sources. The changes in five key pollutants (PM_2.5_, PM_10_, CO, SO_2_, and NO_2_ reported by the China National Environmental Monitoring Center) in eight cities during APEC are shown in Fig. S11. The average changes showed clear spatial variations with larger reductions in Beijing and cities located southwest of Beijing (e.g., Langfang, Baoding, and Shijiazhuang, Fig. S12d). The average reductions of PM_2.5_ and PM_10_ in these cities were 41–68% and 46–64%, respectively, which is consistent with the 50–61% reduction in PM_1_ measured in this study. Gaseous pollutants showed smaller reductions compared to PM, with 25–40% for CO, 27–33% for NO_2_, and 27–38% for SO_2_. These results highlight the importance of synergic control of a range of precursors of secondary aerosol in mitigation of PM pollution in China.

Secondary sulfate and nitrate showed relatively constant 260 m/ground ratios (*R*_H/L_, 0.6–1.0) among different episodes (Fig. 1C) indicating the similarity of secondary inorganic species at different heights in the city. This highlights the regional character of sulfate and nitrate and suggests that measurements of secondary species at the ground site are representative of regional conditions. While SOA shows a similar variation of *R*_H/L_, the ratio is higher than that of SIA indicating the importance of SOA at 260 m. POA and chloride however showed very different *R*_H/L_ before and during APEC. The average *R*_H/L_ during APEC2 is 0.2 and 0.4 for POA and chloride, respectively, which is much lower than before APEC. As shown in Fig. 1A, aerosol composition at 260 m was similar before and during APEC despite the emission controls. This observation suggests that primary aerosol species at high altitudes are mainly from regional transport and show similar reductions due to emission control as secondary species do, whereas the concentration of primary species at the ground site largely depends on local emission controls. Our results also have significant implications, highlighting the importance of aerosol particle composition measurements at high altitudes for investigating the effects of regional transport.

### Size Distributions

**Figure 2:**
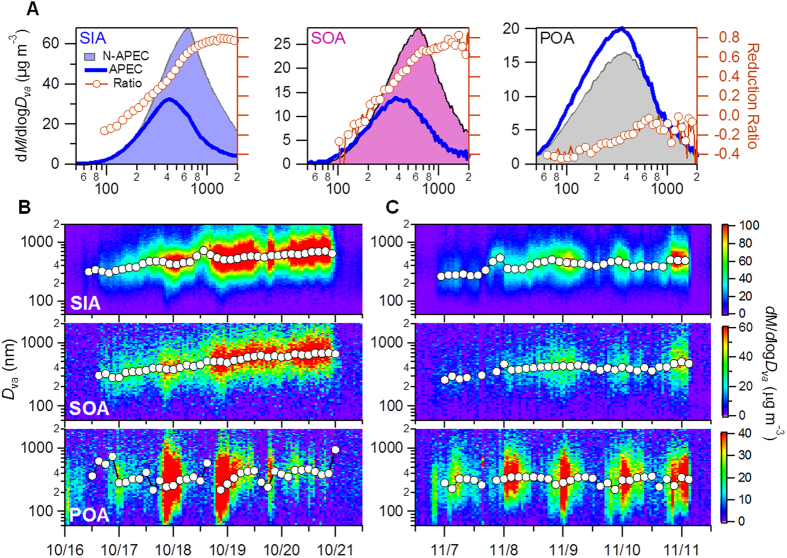
Size distributions of primary and secondary aerosols. (**A**) Average mass-weighted size distributions of SIA, SOA and POA before (15 October–3 November, N-APEC) and during APEC (3–12 November). The reduction ratio ((N-APEC–APEC)/N-APEC) is shown on the right axis. (**B,C**) show the size evolution of SIA, SOA, and POA during Ep1 and APEC2 (see Fig. 1). Solid circles show the peak diameters from the log-normal fit of each size distribution.

SIA and SOA showed large accumulation modes peaking at ~600 nm in vacuum aerodynamic diameter (*D*_va_) before APEC while POA showed a broader size distribution peaking at ~400 nm (Fig. 2). Secondary aerosol dominated accumulation mode particles accounting for more than 80% of the mass in particles larger than 400 nm. The POA contribution increased rapidly up to 80% when the size drops to ~70 nm. The size distributions of SIA and SOA showed significant changes during APEC. While SIA and SOA above 200 nm showed a gradual reduction as a function of particle size by up to ~80% at *D*_va_ > 600 nm, below 200 nm they showed little change and even slight increases (e.g., for SOA) (Fig. 2A). In contrast, POA showed an overall increase across different size ranges during APEC. The size distribution changes for primary and secondary aerosols are further supported by comparing them during the five episodes in this study (Fig. S13B). Although the size distributions varied greatly among different episodes, secondary aerosol overall showed a significant reduction in accumulation mode and primary aerosol showed an increase below 400 nm during APEC. The change of size distributions is consistent with the OA composition change that showed increases for all primary OA components (HOA, COA and BBOA) and corresponding decreases for SOA (SV-OOA and LV-OOA). The average POA contribution increased from 42% to 64% during APEC associated with a decrease of SOA from 57% to 36% (Fig. S13A). Consistently, the size-segregated aerosol samples collected by an eight-stage Andersen cascade impactor also showed a significant reduction above 0.43 μm (~30–60%) for secondary sulfate and nitrate at both the ground site and 260 m during APEC, while chloride showed much less reduction and even an increase for some size ranges (Fig. S14).

A more detailed evolution of the size distributions showed a clear growth of SIA and SOA in particle size during Ep1 (Fig. 2B). The peak size increased from ~300 nm to ~700 nm over a four-day period, while POA didn’t show a similar growth with the peak size remaining at ~300 nm. A very different size evolution was observed during APEC (Fig. 2C). Although SIA and SOA showed a slow growth from ~300 nm to ~500 nm during the first two days, they remained at ~400–500 nm over the next two days. However, POA showed a similar peak size to that before APEC, ~300 nm. These results indicate that regional emission controls have a large impact in suppressing particle growth by secondary species. Note that the relatively dry conditions during APEC (39 ± 17% versus 53 ± 23% before APEC) may also have played a critical role in suppressing the growth of aerosol particles.

### Meteorological Effects

**Figure 3:**
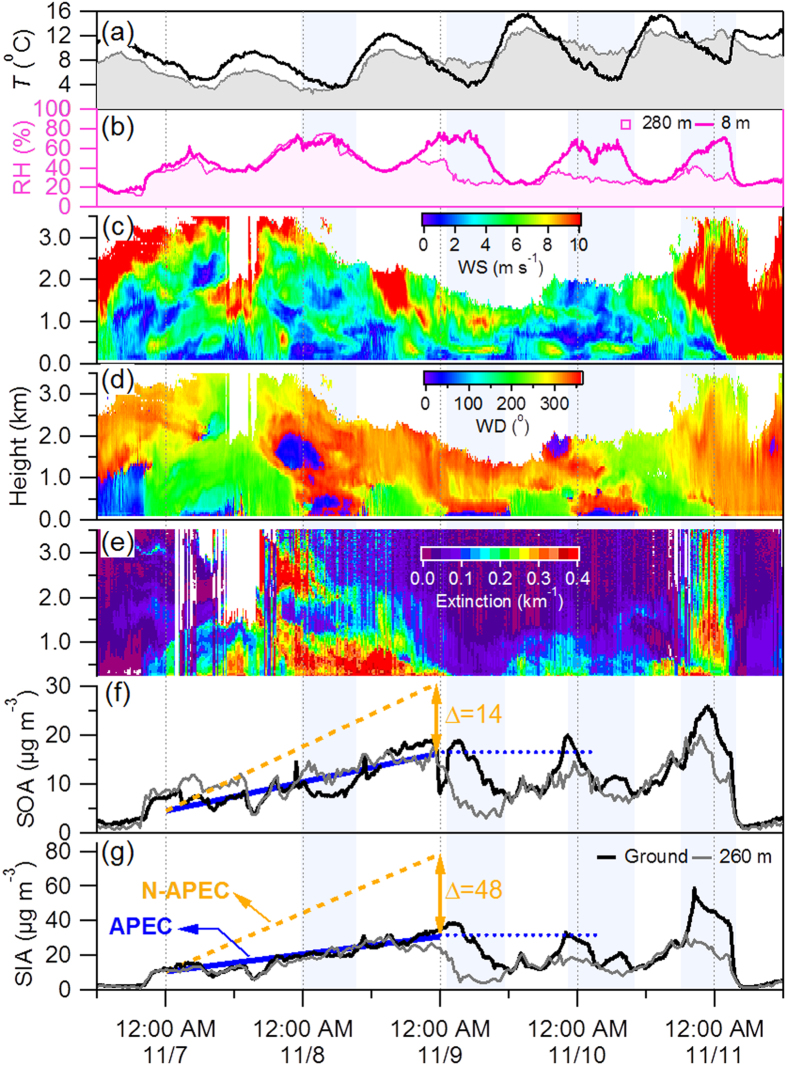
Evolution of meteorological parameters and secondary aerosol during APEC. Time series of (**a**) temperature and (**b**) relative humidity at 260 m and at the ground site, vertical profiles of (**c**) wind speed, (**d**) wind direction, and (**e**) extinction coefficient, and (**f,g**) SOA and SIA at 260 m and the ground site. The solid blue lines represent the accumulation of SIA and SOA, respectively during APEC, and the two orange dash lines refer to the estimated accumulation of SIA and SOA without emission controls. The differences of SOA and SIA with and without emission controls after two days are 14 and 48 μg m^−3^, respectively. The dashed blue lines indicate a roughly stable variation of the maximum concentration of SOA and SIA.

Figure 3d shows that the wind direction switched from northeasterly to southerly below 1 kilometer at 20:00 on 6 November which led to a rapid increase of SIA from ~2 μg m^−3^ to ~10 μg m^−3^ and SOA from ~3 μg m^−3^ to ~7 μg m^−3^ in two hours. SIA and SOA showed almost linear increases during the following two days. Owing to the regional emission controls, the growth rates of SIA and SOA were significantly suppressed compared to the episode of 16–18 October which had similar meteorological conditions (Fig. S15). The average growth rates of SIA and SOA were 0.42 and 0.25 μg m^−3^ hr^−1^, respectively, which is less than half of those observed before APEC (1.42 and 0.55 μg m^−3^ hr^−1^, respectively). As a result, SIA and SOA were reduced by 48 and 14 μg m^−3^, respectively, over a two day period due to the emission controls.

The evolution of air pollution during the later stage of the APEC episode (9–11 November) was strongly associated with the mountain-valley breeze that occurs routinely at midnight and brings a change of wind direction from south to northwest/northeast (Fig. 3d). The penetration of clean air mass from the upper layer (>1 km) led to a rapid decrease in SIA and SOA (shaded areas in Fig. 3d). Such a unique cycle of mountain-valley wind greatly reduced PM_2.5_ concentration by a factor of more than two from >75 μg m^−3^ at night to <40 μg m^−3^ during daytime. As a result the daily average PM_2.5_ concentration met the China ambient air quality standard (75 μg m^−3^ for a 24 hour average) and contributed to the achievement of “APEC Blue”. A similar meteorological pattern occurring during the episode of 18 October before APEC also led to a reduction of PM_2.5_ by 70–80 μg m^−3^ during daytime.

SIA and SOA started to build up immediately when the mountain-valley breeze disappeared (~12:00 PM) and the wind direction switched to southerly. In fact, the daily maximum concentrations of SIA and SOA during 9–11 November were relatively similar indicating that regional transport from the south with higher PM levels (Fig. S16) was still a major source of secondary aerosol in Beijing during APEC. The PM_2.5_ concentrations in cities along the Taihang Mountains and near Beijing (e.g., Langfang, Tianjin, and Baoding) showed similar diurnal variations driven by the mountain-valley breeze, but it had almost no impact on cities outside this region (e.g., Dezhou and Cangzhou in Shandong province) (Fig. S16). Our results demonstrate that the mountain-valley breeze can substantially reduce particulate matter pollution over a specific region for a short time, but does not significantly affect the accumulation of secondary aerosol over the entire highly polluted region. This also suggests that the dramatic reduction of secondary aerosol during APEC was primarily due to regional emission controls.

### Conceptual Framework for Primary and Secondary Aerosol Evolution

Based on the results above, we propose a conceptual framework for primary and secondary aerosol evolution with and without emission controls. This framework is derived from the comparison of two episodes before and during APEC, i.e., Ep1 and APEC2 which showed relatively similar synoptic system over a regional scale (Fig. S17). The two episodes both started with a rapid increase of all aerosol species for two hours which formed a regional background (see Fig. 3 and Fig. S8 for details). The regional background conditions during Ep1 and APEC2 were similar, determined as 0.3, 10.4 and 4.5 μg m^−3^ for BC, SIA and SOA, respectively. BC was apportioned into local emissions and regional transport using a linear regression model which was described in detail in supporting information. While local BC and POA varied similarly day by day, SIA and SOA accumulated approximately linearly in the first two days under stagnant meteorological conditions (wind speed <~2 m s^−1^) and then remained relatively constant except for a short period of reduction due to cleaning by the mountain-valley wind. The variations of secondary aerosol were dramatically different from local primary aerosol indicating their very different sources. Considering the similar accumulation rates of PM_2.5_ in Beijing and surrounding regions and also the progression of the PM_2.5_ peak from the south to the north, we conclude that secondary aerosol at our sampling site was predominantly contributed by regional transport. Further support for this conclusion is provided by the source footprint of air masses identified using the FLEXPART model which showed the dominant source regions to the south and southwest of Beijing during the first two days in both episodes (Fig. S19).

**Figure 4:**
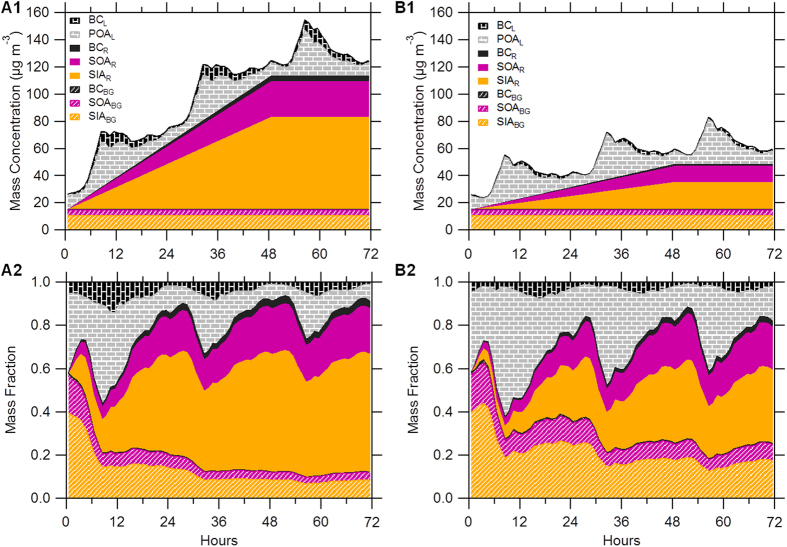
A conceptual framework on primary and secondary aerosol evolution. The 72 hr evolution of mass concentrations and mass fractions of primary and secondary aerosol without emission controls before APEC (A1 and A2) and with the emission controls during APEC (B1 and B2). The evolution of primary species starts from noontime with te lowest concentrations in a day. SIA_BG_, SOA_BG_, and BC_BG_ refer to the regional background concentrations of secondary inorganic aerosol, secondary organic aerosol, and black carbon. SIA_R_, SOA_R_, and BC_R_ are the concentrations from regional transport which are calculated based on the accumulation rates during the episodes of 16–17 October and 7–8 November, respectively. BC_L_ and POA_L_ refer to the local sources that were calculated from the diurnal profiles of POA (HOA, COA, and BBOA) and BC (regional background and transport were excluded) before and during APEC. A2 and B2 show the evolution of the mass fraction of aerosol species from different sources.

Although local chemical production and boundary layer dynamics also affected the variations, they did not change the overall increase in secondary aerosol. A linear fit was performed on the SIA and SOA mass concentrations during the accumulation stage for the two episodes to reduce the influence of local variables on the evolution of SOA. We propose a conceptual framework for aerosol evolution with and without emission controls by segregating it into three parts, i.e., regional background, accumulation from regional transport, and local sources. The local sources and regional transport with and without emission controls were derived from the variations of primary (POA and BC) and secondary (SIA and SOA) aerosol during Ep1 and APEC2, respectively. Because the local POA and BC emissions show pronounced diurnal variations, aerosol evolution represents an upper limit when it starts from midnight with the highest primary aerosol concentration (Fig. 4) and a lower limit when it starts from noontime with the lowest concentration (Fig. S19). As seen in Fig. 4A1, the total PM_1_ mass increased rapidly and reached 75–96 μg m^−3^ in 24 hours and up to 120–145 μg m^−3^ in two days. Regional transport played a greater role during the evolution of PM with the contribution increasing from 4–8% to 51–65% in 24 hours and up to 66–80% in two days. Owing to the emission controls during APEC, the total PM_1_ mass built up much more slowly reaching 43–59 μg m^−3^ in 24 hours and 60–76 μg m^−3^ in two days, and the contribution of regional transport increased from 2–3% to 28–39% and 44–57%, respectively (Fig. 4B). Our results suggest that regional transport plays a significant role in causing PM pollution during haze episodes in Beijing. This is consistent with previous studies that show a large contribution of PM_2.5_ during haze episodes in Beijing from regional transport (often more than 50%)^9^,^15^. By comparing the evolution of aerosol species with and without emission controls, we find that the emission controls during APEC reduced PM_1_ by 38–43% in 24 hours and by 43–52% in two days, mainly by suppressing secondary formation regionally.

## Implications

We demonstrate the response of aerosol composition, size distributions, and source contributions in Beijing to emission controls during APEC based on comprehensive measurements at both ground level and at a height of 260 m in urban Beijing. We observed large reductions of secondary aerosols during APEC, of 61–67% and 51–57% for SIA, and of 55% and 37% for SOA at 260 m and the ground site, respectively, whereas primary aerosols at ground level did not change in the same way. This large reduction of secondary aerosol is closely linked to the corresponding reduction of precursors over a regional scale, which suppresses the formation and growth of secondary aerosol by a factor of 2–3. Our results demonstrate that the achievement of “APEC Blue” is largely a result of significant reductions of secondary aerosol due to emission controls, although the mountain-valley breeze circulation also played a role. A proposed conceptual framework on the evolution of primary and secondary aerosol further highlights the dominant role of regional transport in haze formation even under stagnant meteorological conditions. Regional transport can contribute up to 66–80% of the total PM mass over a two day period and still as much as 44–57% with the emission controls during APEC. Our results provide direct evidence that reducing the precursors of secondary aerosol over regional scales is the most effective approach to suppress the formation of secondary particulates and hence to mitigate particulate matter pollution in Beijing. However, the fact that bulk aerosol composition at 260 m was similar before and during APEC suggests that the synergistic control of a range of precursors had little effect on regional aerosol composition.

## Methods

### Sampling

The measurements were conducted between 15 October and 13 November 2014 at the Institute of Atmospheric Physics, Chinese Academy of Sciences, an urban site in the north of Beijing. The composition of size-resolved non-refractory submicron aerosol (NR-PM_1_) at the ground site (~4 m) was measured with an HR-AMS. Collocated real-time measurements include black carbon (BC), particle light extinction, and a range of gaseous species including CO, O_3_, NO, NO_y_, and SO_2_. In addition, we deployed an ACSM for the real-time measurements of NR-PM_1_ aerosol species at a height of 260 m at the same location^23^. Size-segregated samples at 260 m and at near ground level were also collected twice per week using two eight-stage non-viable Andersen cascade impactors (Series 20–800, Thermo Scientific) from 30 October to 27 November, 2014. All filter samples were collected using pre-combusted quartz filters (6 h in 450 °C in a muffle furnace).

The vertical profiles of back scattering coefficients at 532 nm and 1064 nm were measured using a dual-wavelength depolarization Lidar. The extinction coefficients were retrieved using the Fernald inversion method^24^. Wind profiles between 100 m and 5000 m were measured using a Doppler Wind Lidar (Windcube 200, Leosphere, Orsay, France). The radial wind speed along four cardinal geographical directions was measured sequentially and the wind sector was then reconstructed. In addition, meteorological parameters including wind speed, wind direction, relative humidity and temperature were obtained at 15 heights on the Beijing 325 m meteorological tower. The detailed instruments and sampling are given in supporting information.

### Chemical Analysis

The size-segregated samples collected at two heights were analyzed for water-soluble ions. An aliquot of each one-eighth sample was sonicated in 10 mL deionized-distilled (DD) water for 60 minutes. The solution was then filtered with 0.45 μm Acrodisc syringe filters. The anions (F^−^, Cl^−^, NO_3_^−^, SO_4_^2−^) and cations (NH_4_^+^) were analyzed by two ion chromatographs (DIONEX, ICS-3000) equipped with separation columns of AS11–HC and CS12A, and guard columns of AG11–HC and CG12A for anion and cation, respectively.

### AMS Data Analysis

The HR-AMS data was analyzed for the mass concentration and size distributions of NR-PM_1_ species using standard AMS data analysis software (SQUIRREL v1.56D). Because aerosol particles were dry and they were only slightly acidic, an universal collection efficiency (CE) of 0.5^25^ was introduced to account for the incomplete detection of aerosol species mainly due to particle bounce from the vaporizer^26^,^27^. The default relative ionization efficiencies (RIEs) which is 1.4 for organics, 1.2 for sulfate, 1.1 for nitrate, and 1.3 for chloride except ammonium (5.0 determined from pure ammonium nitrate) was used. The PM_1_ (NR-PM_1_ + BC) correlates well with the PM_2.5_ (r^2^ = 0.88) and the ratio of PM_1_/PM_2.5_ (0.70) is overall consistent with that reported in previous studies in Beijing^5^,^28^. The results indicate that CE = 0.5 is reasonable for this study.

The high resolution mass spectra (HRMS) of HR-AMS were analyzed with the PIKA software (version 1.15D)^29^. The ion fragments that can be segregated into different ion categories including C_x_H_y_^+^, C_x_H_y_O^+^, C_x_H_y_O_2_^+^, C_x_H_y_N_p_^+^, H_x_O^+^, etc were separated and quantified. The elemental ratios of OA including hydrogen-to-carbon (H/C), oxygen-to-carbon (O/C), nitrogen-to-carbon (N/C), and organic mass-to-carbon (OM/OC) were then determined using the elemental analysis technique^30^.

The ACSM data were analyzed for the mass concentrations of NR-PM_1_ species using ACSM standard data analysis software (v 1.5.3.0). The detailed procedures for the data analysis have been described in Ng *et al.*^31^ and Sun *et al.*^28^. Similar to our previous study^5^, the CE of 0.5 was used in this study because the particle acidity, aerosol composition, and particle phase water all have minor influences on CE in this study^27^,^32^.

Positive matrix factorization (PMF)^33^ was performed on the HRMS of OA to resolve distinct OA factors. The pre-treatment of data and error matrices before the PMF analysis are detailed in Ulbrich *et al.*^34^. The mass spectral profiles and time series of OA factors were evaluated following the steps recommended by Zhang *et al.*^35^. A six-factor solution was chosen, which includes hydrocarbon-like OA (HOA), biomass burning OA (BBOA), semi-volatile oxygenated OA (SV-OOA), low-volatility OOA (LV-OOA), and two cooking OA (COA). PMF analysis was also performed on the unit mass resolution of organic mass spectra of ACSM between *m/z* 12–120. The PMF results were then evaluated using the same procedures as those for PMF-AMS, and two OA factors including a primary HOA and a secondary OOA were resolved. To compare better with 260 m, the four primary OA factors, i.e., HOA, two COA, and BBOA were recombined into one POA factor, and the two OOA factors were combined into one SOA factor at ground site. A more detailed description of the PMF analysis is given in the supplementary material.

## Additional Information

**How to cite this article**: Sun, Y. *et al.* “APEC Blue”: Secondary Aerosol Reductions from Emission Controls in Beijing. *Sci. Rep.* **6**, 20668; doi: 10.1038/srep20668 (2016).

## Supplementary Material

Supplementary Information
