# Supplementary material for: “APEC Blue”: Secondary Aerosol Reductions from Emission Controls in Beijing
Source: Sci Rep. 2016 Feb 18;6:20668. doi: 10.1038/srep20668 (PMC4758222; doi:10.1038/srep20668)
Supplement: Supplementary Information [file srep20668-s1.pdf]

Supplementary Information

"APEC Blue": Secondary Aerosol Reductions from Emission Controls in Beijing

Yele Sun[1,*], Zifa Wang[1], Oliver Wild[2], Weiqi Xu[1], Chen Chen[3,1], Pingqing Fu[1], Wei Du[1], Libo Zhou[1], Qi Zhang[4], Tingting Han[1], Qingqing Wang[1], Xiaole Pan[5], Haitao Zheng[1,6], Jie Li[1], Xiaofeng Guo[1], Jianguo Liu[6], Douglas R. Worsnop[7]

[1]State Key Laboratory of Atmospheric Boundary Layer Physics and Atmospheric Chemistry, Institute of Atmospheric Physics, Chinese Academy of Sciences, Beijing 100029, China

[2]Lancaster Environment Centre, Lancaster University, Lancaster LA1 4YQ, UK

[3]College of Applied Meteorology, Nanjing University of Information Science and Technology, Nanjing 210044, China

[4]Department of Environmental Toxicology, University of California, 1 Shields Ave., Davis, CA 95616, USA

[5]Research Institute for Applied Mechanics, Kyushu University, Fukuoka 819-0395, Japan

[6]Anhui Institute of Optics and Fine Mechanics, Chinese Academy of Sciences, Hefei 230031, China

[7]Aerodyne Research, Inc., Billerica, Massachusetts 01821, USA

*Corresponding author email: sunyele@mail.iap.ac.cn; Phone: +86-10-8202-1255

1. Instrumentation

An Aerodyne High Resolution Time-of-Flight Aerosol Mass Spectrometer (abbreviated as HR-AMS hereafter) was used to for the real-time measurement of size-resolved non-refractory submicron aerosol (NR-PM_1) composition including organics, sulfate, nitrate, ammonium, and chloride [1]. The HR-AMS was operated with two ion optical modes, i.e., V-mode with more sensitive signals and W-mode with higher mass resolution in this study. The V-mode and W-mode alternated every 5 minutes. Under V-mode operation, the HR-AMS cycled through the mass spectrum (MS) mode and the particle time-of-flight (PToF) mode every 10 s. A $PM_{2.5}$ cyclone (URG-2000-30EN, URG Corporation) was supplied in front of the sampling line to remove coarse particles larger than 2.5 μm. After passing a silica gel dryer, the ambient air was sampling into the HR-AMS at a flow of ~0.1 L/min. The HR-AMS was calibrated for ionization efficiency (IE) and particle sizing before the measurements following the standard protocols [2,3]. The 5 min detection limits (DLs) of NR-PM_1 aerosol species determined as 3 times the standard deviations (3σ) of mass concentrations of particle-free ambient air are 17, 10, 1.6, 1.4, and 4 ng/m^3 respectively for V-mode and 30, 35, 26, 4.9, and 3.2 ng/m^3 respectively for W-mode, which are close to the values reported in previous studies [1,4]. Because HR-AMS cannot detect refractory black carbon (BC), a 2-wavelength Aethalometer (Model AE22, Magee Scientific Corporation) was used to measure BC in $PM_{2.5}$ with a time resolution of 5 min.

A Cavity Attenuated Phase Shift extinction monitor (CAPS PM_{ext}, Aerodyne Research Inc.) was used to measure the light extinction (630 nm) of dry fine particles at a time resolution of 1 second. The CAPS PM_{ext} measures the phase shift (ϑ) of a distorted waveform of the modulated light from a light emitting diode (LED) in the sample cell with two high reflectivity mirrors and the light extinction is determined based on its relationship with ϑ [5]. In addition, a CAPS NO_2 monitor utilizing the same technology as the CAPS PM_{ext} was used to measure the ambient NO_2 at a time resolution of 1 second [6]. Compared to the conventional commercially available

chemiluminescence (CL)-based method, the CAPS NO_2 monitor that essentially has no interferences with nitrogen containing species has significant improvements in terms of sensitivity and accuracy. Other gaseous pollutants including CO, O_3, NO, NO_y, and SO_2 were measured by a suite of commercial gas analyzers from Thermo Scientific.

In addition to the real-time online measurements, $PM_{2.5}$ quartz filter samples were also collected at the ground site using a high-volume air sampler (flow rate: 1.0 m^3 min^{-1}). One daytime sample (7:00 – 18:00) and one nighttime sample (18:00 – 7:00) were collected for each day. After sampling, the filters were stored in a pre-combusted glass jar (150 ml) with a Teflon-lined screw cap at –20°C until analysis. The size-segregated samples at two heights, i.e., the ground site and 260 m were also collected using two eight-stage non-viable Andersen cascade impactor (Series 20 – 800, Thermo Scientific) from 30 October to 27 November 2014. The cutoff points of the size-segregated samples are 0.43, 0.65, 1.1, 2.1, 3.3, 4.7, 5.8 and 9.0 μm, respectively. In this study, two samples per week were collected. In addition, field blank samples were also collected at the beginning and the end of the sampling campaign by placing pre-baked blank filters onto the samplers for about 10 min without sampling ambient air. Both $PM_{2.5}$ and size-segregated quartz filters (Pallflex) were pre-combusted for 6 h at 450°C in a muffle furnace.

A dual-wavelength depolarization Lidar (Model: L2S-SM II) developed by the National Institute for Environmental Studies (NIES) was used to measure the vertical profiles of back scattering coefficient at 532 nm with 15-min intervals and 30-m height resolution [7]. The extinction coefficient was retrieved using the Fernald inversion method [8] with a lidar ratio (extinction-to-backscatter ratio) of 50 sr. A Doppler Wind Lidar (Windcube 200, Leosphere, Orsay, France) was deployed at the same location to obtain the wind profiles from 100 m to 5000 m with a spatial resolution of 50 meters and a time resolution of 10 minutes. The wind lidar was operated at the near-IR wavelength (1.54 μm) with the pulse energy being 100 μJ. The radial wind speed along four cardinal geographical directions was measured

sequentially and the wind sector was then reconstructed. The accuracy for the measurements of wind speed and wind direction is 0.5 m s^{-1} and 1.5°, respectively. The detailed principles of the wind lidar are given in Ruchith et al. [9]. Additional 15 heights (8, 15, 32, 47, 63, 80, 102, 120, 140, 160, 180, 200, 240, 280, and 320 m) of meteorological parameters including wind speed, wind direction, relative humidity and temperature were also obtained from the measurements on the 325 m meteorological tower.

The non-refractory submicron aerosol composition was also measured by the ACSM at 260 m on the Beijing 325 m meteorological tower. The ambient air was drawn inside the container where the ACSM was located at a flow rate of 3 L/min, of which ~0.1 L/min was sub-sampled into the ACSM. Similar to AMS setup, a $PM_{2.5}$ cyclone (Model: URG-2000-30ED) was supplied in front of the sampling line to remove coarse particles larger than 2.5 μm, and a silica gel dryer was used to dry aerosol particles before entering the ACSM. The ACSM was operated at a scanning rate of 500 ms amu^{-1} for the mass spectrometer from *m/z* 10 – 150. The data was saved every two cycles by alternating ambient air and filtered air, leading to a time resolution of ~ 5 min. The detailed operations and calibrations of the ACSM are given in Ng et al. [10] and Sun et al. [11].

2. PMF analysis of HR-AMS and ACSM datasets

Positive matrix factorization (PMF) [12] was performed on the HRMS of HR-AMS to resolve potential OA factors from different sources. The data and error matrices were pre-treated following the procedures detailed in Ulbrich et al. [13] before the PMF analysis. Ions with S/N ratio < 0.2 were removed from the HRMS data and error matrices. The "weak" ions with S/N between 0.2 and 2 were downweighed by increasing their errors by a factor of 2 [13,14]. In addition, the errors for H_2O^+, HO^+, O^+, CO^+, and CO_2^+ were increased by a factor of 2.2 because they were all scaled based on its relationship to CO_2^+ and may introduce additional weights in the PMF analysis [13]. The mass spectral profiles and time series of OA factors were evaluated following the steps recommended by Zhang et al. [15]. A summary of key PMF diagnostic plots is

presented in Fig. S1 and Fig. S2. Eventually, a 6-factor solution with fPeak = 0 ($Q/Q_{expected}$ = 1.552) was chosen in this study, which includes a hydrocarbon-like OA (HOA), two cooking OA (COA), a biomass burning OA (BBOA), a semi-volatile oxygenated OA (SV-OOA), and a low volatility OOA (LV-OOA). The mass spectral profiles and time series of the six factors are shown in Fig. S3. To better compare with the results from PMF analysis of ACSM organic mass spectra, the four primary OA factors, i.e., HOA, COA, and BBOA were recombined into one POA factor, and the two OOA factors were recombined into one SOA factor.

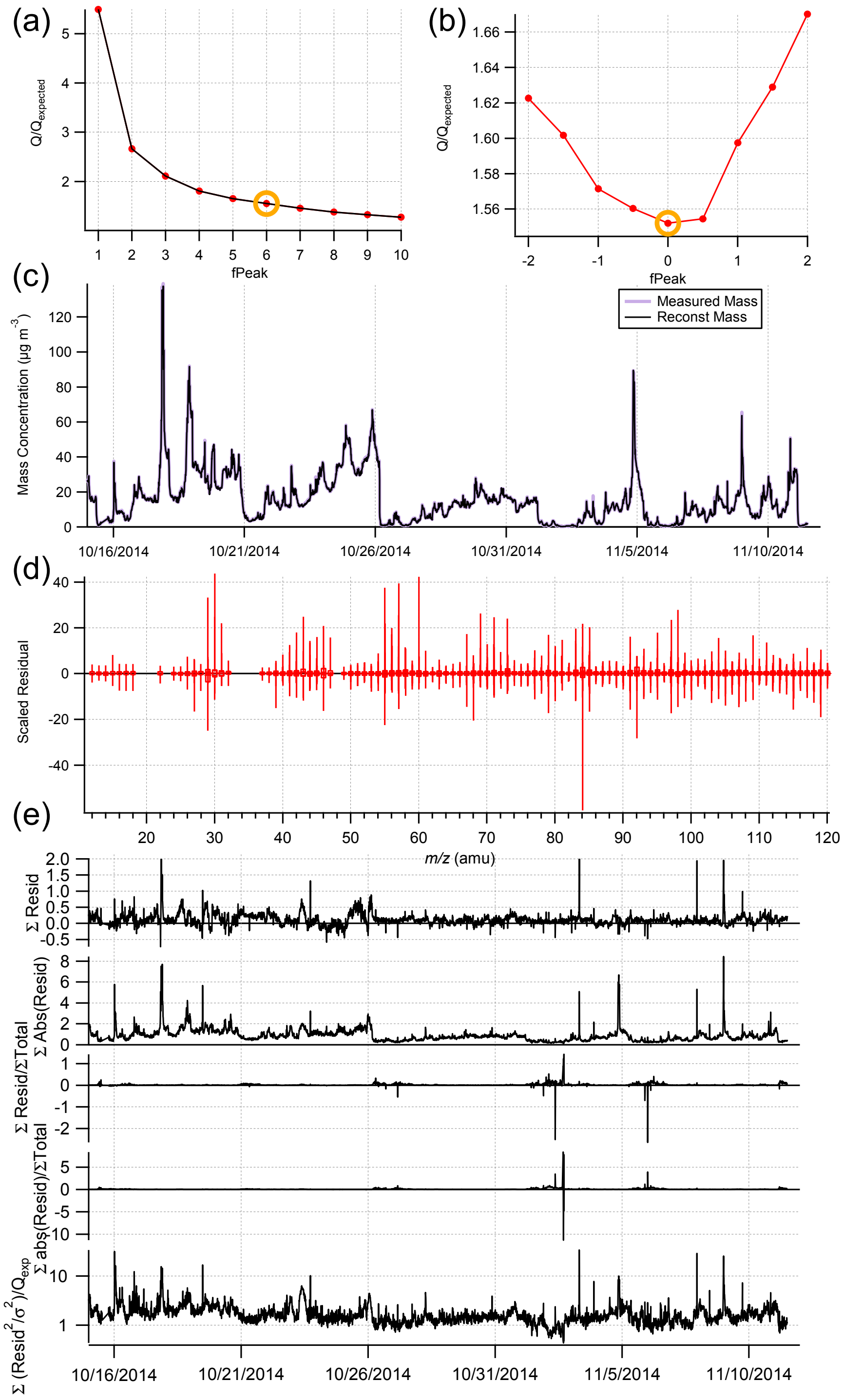

Figure S1. A summary of diagnostic plots for the PMF analysis of HRMS of AMS: (a) $Q/Q_{expected}$ as a function of the number of factors (*p*), (b) $Q/Q_{expected}$ as a function of fpeak values for the 6-factor solution; (c) time series of the measured mass concentration and the reconstructed mass; (d) box plots of residuals for each ion fragment; (e) time series of ΣResidual, ΣAbs(residual), ΣResidual/Total, ΣAbs(residual)/Total, and $Q/Q_{expected}$ values.

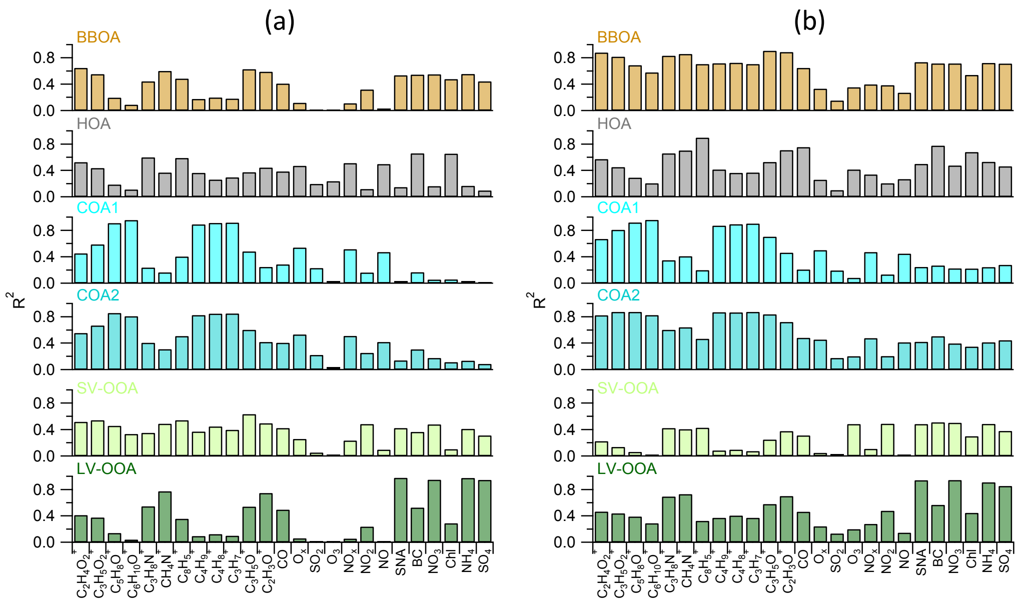

Figure S2. Correlations of six OA factors with external tracers (a) before and (b) during APEC.

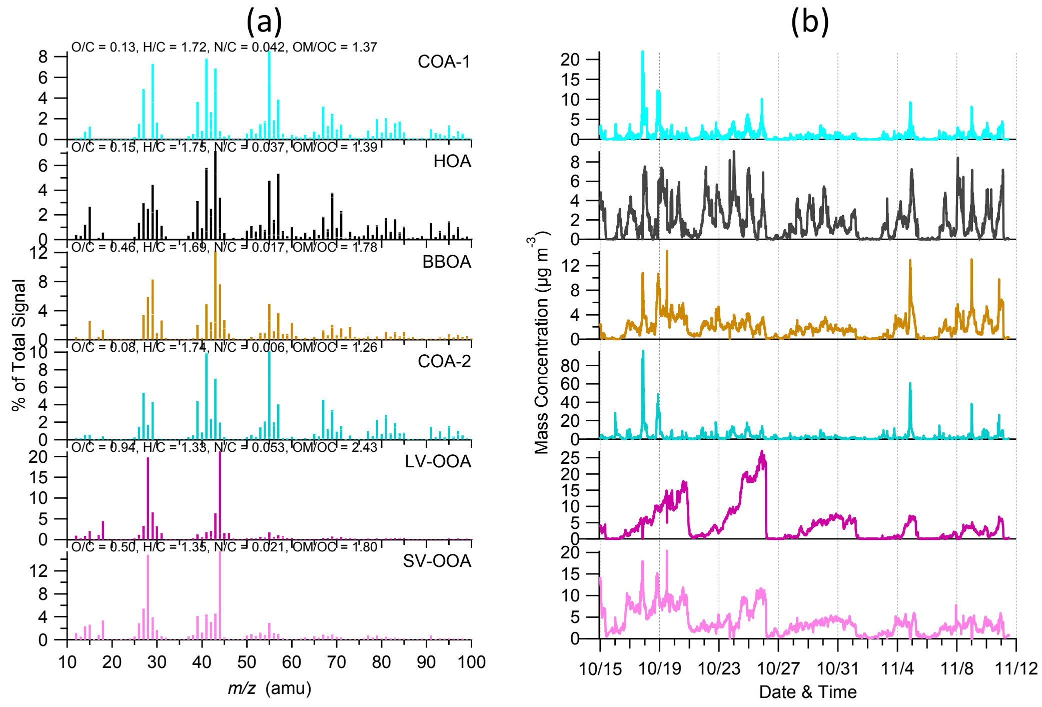

Figure S3. (a) Mass spectral profiles and (b) time series of six OA factors, i.e., two cooking organic aerosol (COA), a hydrocarbon-like OA (HOA), a biomass burning OA (BBOA), a semi-volatile OOA (SV-OOA), and a low volatility OOA (LV-OOA).

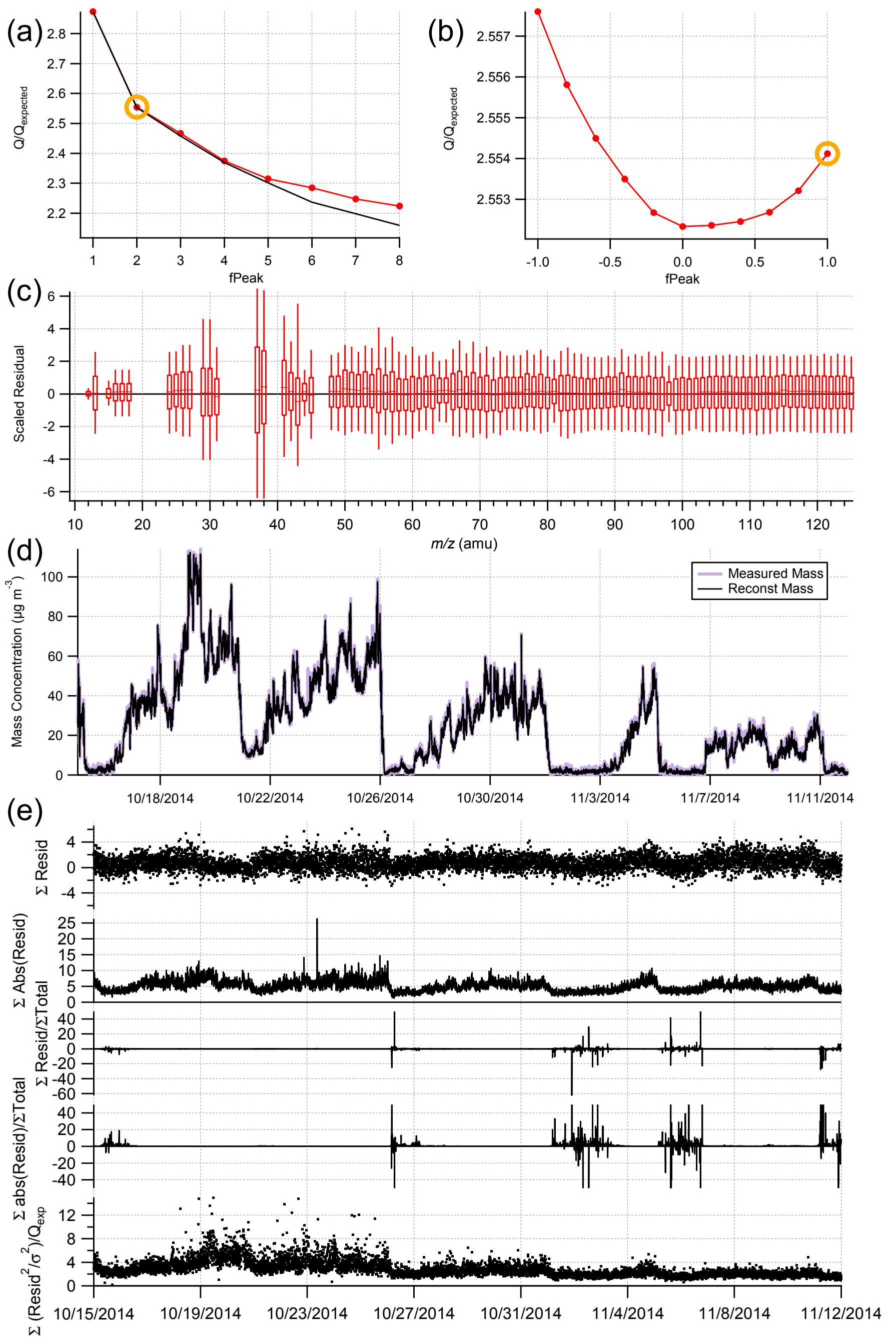

Figure S4. A summary of diagnostic plots for the PMF analysis of ACSM OA mass spectra: (a) $Q/Q_{expected}$ as a function of the number of factors (*p*), (b) $Q/Q_{expected}$ as a function of fpeak values for the 2-factor solution; (c) time series of the measured mass concentration and the reconstructed mass; (d) box plots of residuals for each ion fragment; (e) time series of ΣResidual, ΣAbs(residual), ΣResidual/Total, ΣAbs(residual)/Total, and $Q/Q_{expected}$ values.

The PMF was also performed on the unit mass resolution mass spectra of organic aerosol from the ACSM measurements. The PMF analysis was limited to *m/z* 120 mainly due to large interferences of internal standard of naphthalene at *m/z*'s 127 - 129. The PMF results were further evaluated with an Igor Pro-based PMF Evaluation Tool (PET, v2.04) [13], and the number of PMF factors were determined following the procedures detailed in Zhang et al. [15]. Here, two OA factors including a primary OA (POA) and a secondary OA (SOA) were identified. A summary of key PMF diagnostic plots is given in Fig. S4. The mass spectral profiles and time series of two OA factors are shown in Fig. S5.

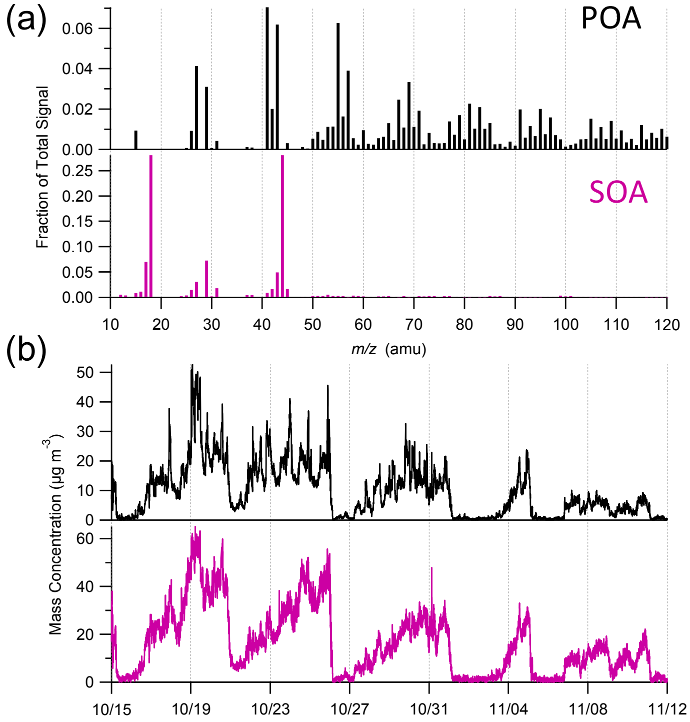

Figure S5. (a) Mass spectral profiles and (b) time series of two OA factors which is POA and SOA.

3. Size distributions of SOA and POA

The size distributions of SOA were derived from that of *m/z* 44 by normalizing the integrated signals of *m/z* 44 between 30-1500 nm to the concentrations of SOA. This method is rationale because *m/z* 44 (mainly CO_2^+) highly correlated with OOA (r^2 =

0.98) [16]. The size distributions of POA were then obtained from the differences between the total OA and SOA. It should be noted that we might slightly overestimate the SOA concentration in small size ranges because ~17% of *m/z* 44 was found to be contributed by POA.

4. Source apportionment of black carbon

The sources of BC were investigated using a linear regression technique. Such a technique was used to characterize the sources of BC relating to different aerosol species in Lanzhou, China [17]. The results showed that more than half of BC was associated with secondary aerosol (sulfate, nitrate, LV-OOA and SV-OOA), which was likely from regional transport. In this study, BC was apportioned into different sources using the following equation:

$$[BC] = a_{HOA}[HOA]+ a_{BBOA}[BBOA]+ a_{COA}[COA]+ a_{SV\text{-}OOA}[SV\text{-}OOA]+ a_{SNA}[SNA] + C \quad (1)$$

where SNA refers to sulfate, nitrate, and ammonium, and *C* indicates the constant value. Because LV-OOA highly correlated with SNA (r^2 = 0.98 and r^2 = 0.94 before and during APEC, respectively), we didn't include LV-OOA into the equation, and the LV-OOA associated with BC will be accounted for by the SNA. For a better quantification of the sources, the BC data before and during APEC was fitted separately, and the results are shown in equation (2) and (3), respectively:

$$[BC]_{Before\ APEC} = 0.85[HOA]+ 0.074[BBOA]+ 0.036[COA]+ 0.084[SV\text{-}OOA]+ 0.043[SNA] + 0.30 \quad (2)$$

$$[BC]_{APEC} = 0.38[HOA]+ 0.0[BBOA]+ 0.030[COA]+ 0.027[SV\text{-}OOA]+ 0.060[SNA] + 0.39 \quad (3)$$

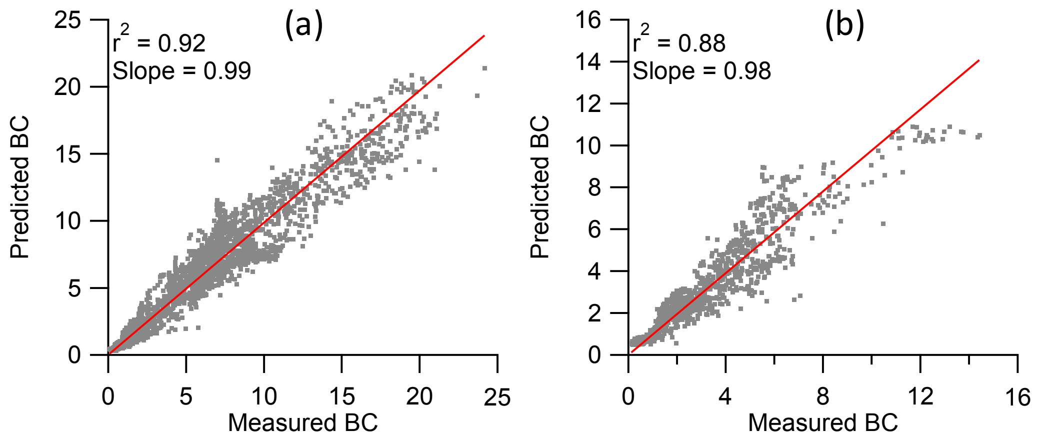

Figure S6. Correlations between predicted BC by Eq. (2) and (3) and measured values (a) before and (b) during APEC.

As indicated in Fig. S6, the reconstructed BC concentrations using Eq. (2) and (3) highly correlated with the measured values before (r^2 = 0.92, slope = 0.99) and APEC (r^2 = 0.88, slope = 0.98). The apportioned BC was then grouped into three different source categories, which are local source emissions (= BC_{HOA} + BC_{COA}+ BC_{BBOA}), regional transport (BC_{SNA}), and background (BC_{C}). The regional background concentration of BC was similar before and during APEC, as a result, the average of 0.35 μg m^{-3} was used in this study. As seen in Fig. S7, BC was dominantly from HOA-related traffic sources, accounting for 45% and 44% of the total before and during APEC, respectively. Also not there were 41% and 38% of BC associating with secondary sources, likely from regional transport.

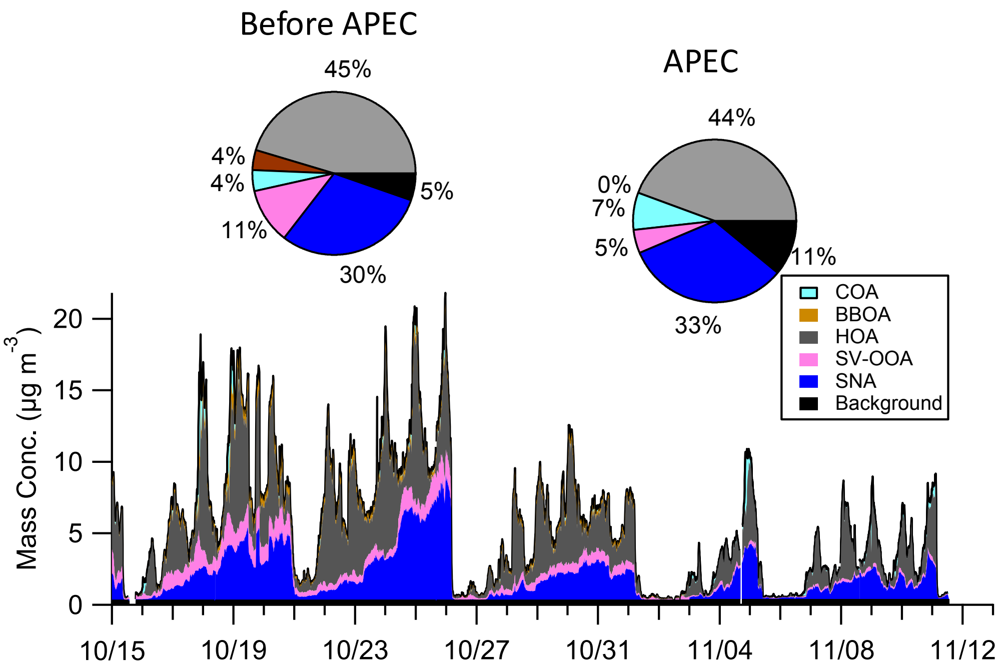

Figure S7. Time series of BC relating to different aerosol species. The two pie charts showed the average apportionment of BC before and during APEC.

5. Calculation of the growth rates of SIA and SOA

The evolution of SIA and SOA that were dominantly from regional transport followed the same patterns before APEC (15:00 16 October – 21:00 18 October, Fig. S8) and during APEC (7 – 9 November, Fig. 3). SIA and SOA increased almost linearly during the first two-day evolution and then remained consistently high during the rest time of the episode. Assuming that SIA and SOA from regional transport were accumulated linearly during the two episodes, and the small variations were mainly caused by the fluctuations of meteorological parameters, a linear fit was then performed on the data of SIA and SOA, and the regression slopes indicated the growth rates of SIA and SOA. Note that mountain-valley breeze from the northeast was also observed to interrupt the evolution of haze episode by reducing daytime PM levels. Therefore the data with clear influences of mountain-valley breeze (00:00 - 14:00, 18 October, and 5:00 – 19:00, 7 November) were excluded from the analysis. The calculated growth rate of SIA and SOA at the ground site were 1.42 µg m^{-3} hr^{-1} and 0.55 µg m^{-3} hr^{-1}, respectively, which were more than twice those during APEC

(0.42 μg m^{-3} hr^{-1} and 0.25 μg m^{-3} hr^{-1}, respectively). The growth rates of SIA and SOA at 260 m were also calculated which were similar to those before APEC. For example, the growth rate of SIA at 260 m was 0.38 μg m^{-3} hr^{-1}, close to 0.41 μg m^{-3} hr^{-1} at the ground site, and the growth rate of SOA at 260 m was 0.47 μg m^{-3} hr^{-1}, which was also close to 0.55 μg m^{-3} hr^{-1} at the ground site. In this study, the growth rates of SIA and SOA calculated from the ground site was used for the concept framework for aerosol evolution.

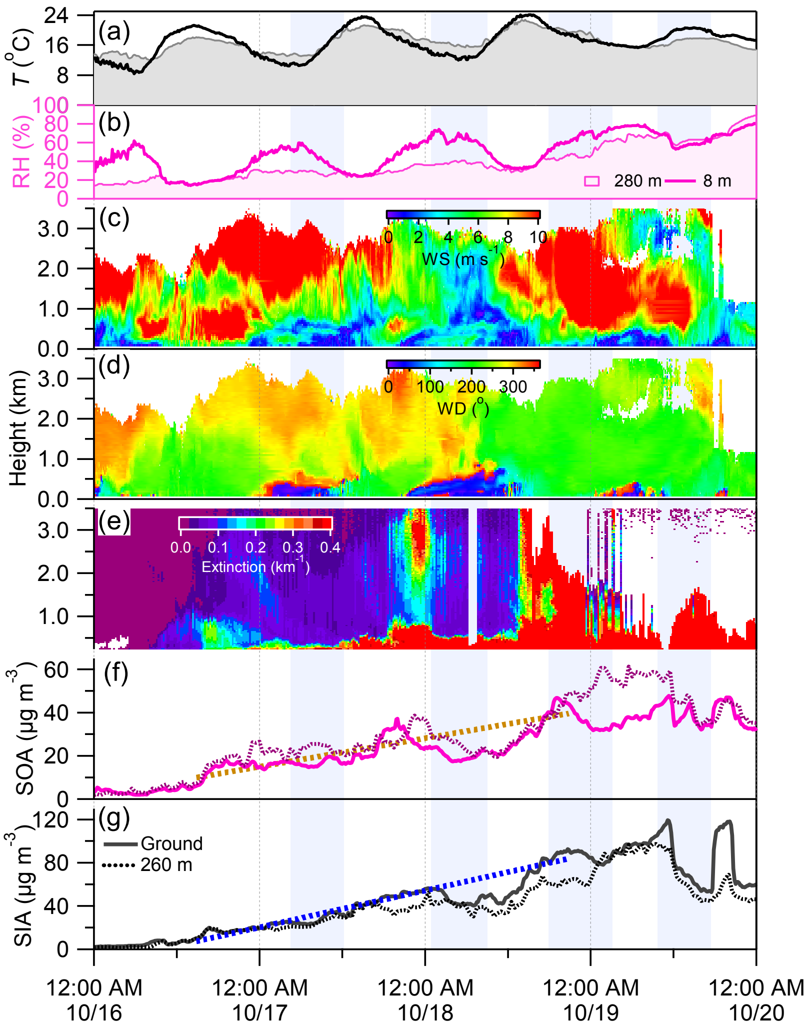

Figure S8. Time series of (a) temperature and (b) relative humidity at 260 m and the ground site, vertical profile of (c) wind speed and (d) wind direction, (e) vertical profile of extinction coefficient, and (f,d) SOA and SIA at 260 m and the ground site. The dash lines represent the predicted accumulation of SIA and SOA, respectively.

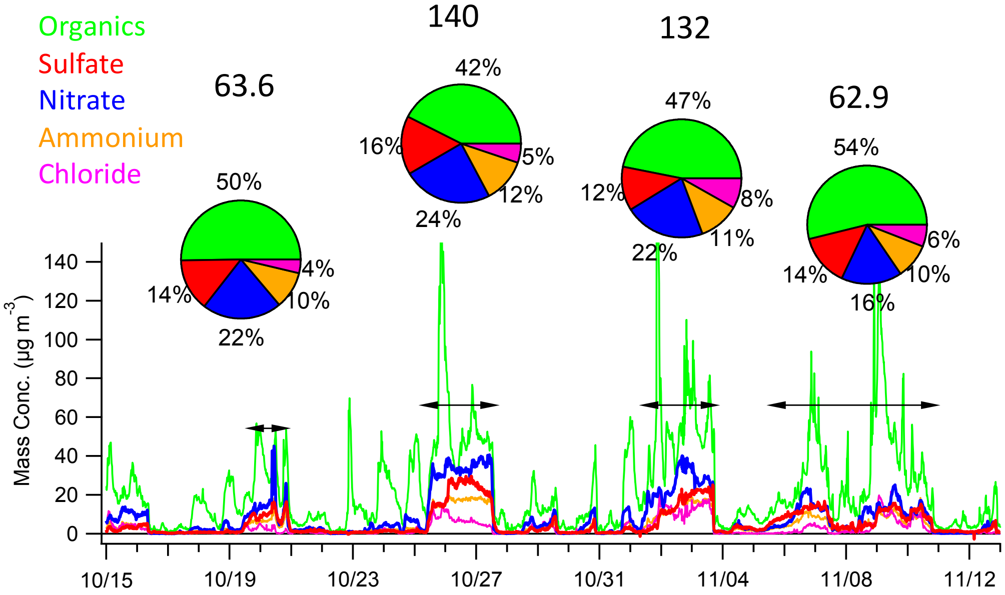

Figure S9. Time series of non-refractory submicron aerosols (organics, sulfate, nitrate, ammonium, and chloride) measured by an Aerodyne aerosol chemical speciation monitor during 15 October to 15 November, 2012. The pie charts show the average chemical composition of four episodes marked in the figure and the number is the average mass concentration for each episode.

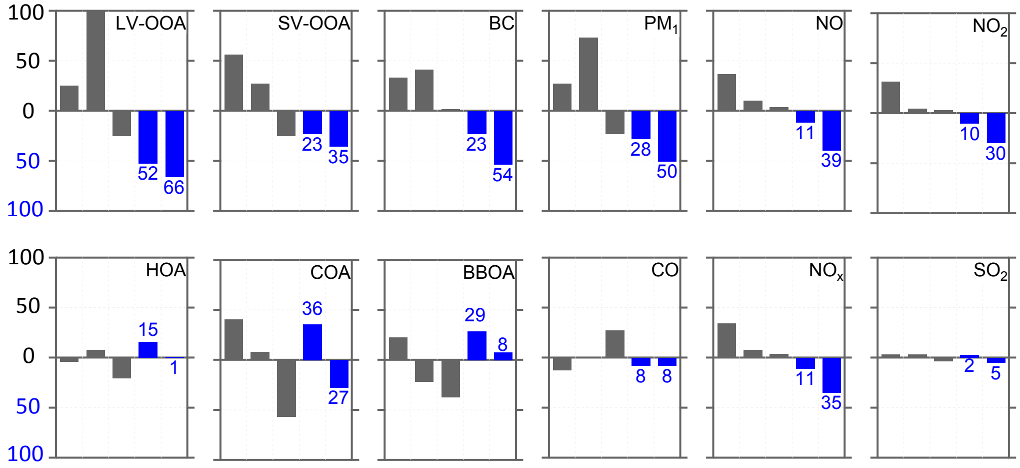

Figure S10. The episode anomalies (%) of aerosol species for each episode referring to the average of five episodes the ground site. The numbers show the change rates of chemical species for the two episodes during the APEC. Secondary species show evidently larger reductions than primary species during the APEC.

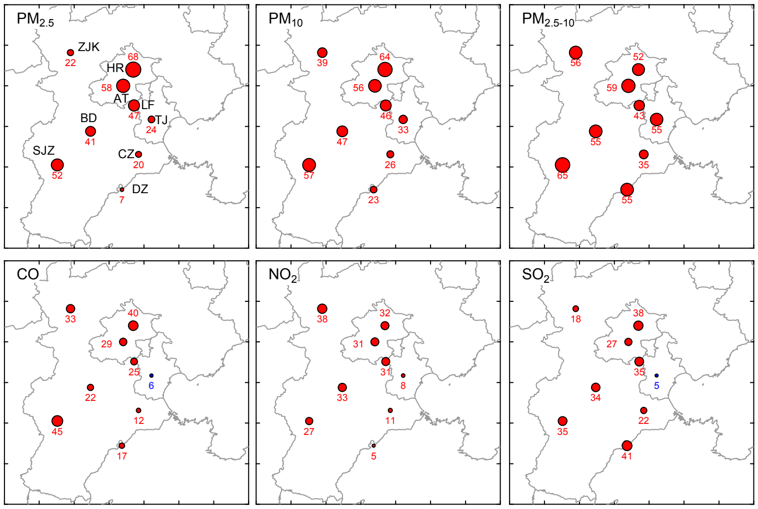

Figure S11. The change rates of various pollutants ($PM_{2.5}$, PM_{10}, $PM_{2.5\text{-}10}$, CO, NO_2, and SO_2) during the episode of APEC (7 – 11 November, APEC2) referring to the average of five episodes (Ep_{Avg}, marked in Fig. 1). The selected sites include an urban site Aotizhongxin (AT) and a rural site Huairou (HR) in Beijing, Tianjing, four cities in Hebei province (Langfang (LF), Baoding (BD), Shijiazhuang (SJZ), and Zhangjiakou (ZJK)) and Dezhou (DZ) in Shandong province. The change rates shown in the figure were calculated as [Ep_{Avg} – APEC2]/ [Ep_{Avg}]. The red numbers indicate reductions and the blue ones indicate enhancements. The $PM_{2.5\text{-}10}$ was calculated as $PM_{10} - PM_{2.5}$. All the data were from the China National Environmental Monitoring Center (CNEMC) (http://113.108.142.147:20035/emcpublish/). The maps were drawn by Igor Pro (version 6.3.7.2, WaveMetrics, Inc., Oregon USA), http://www.wavemetrics.com/.

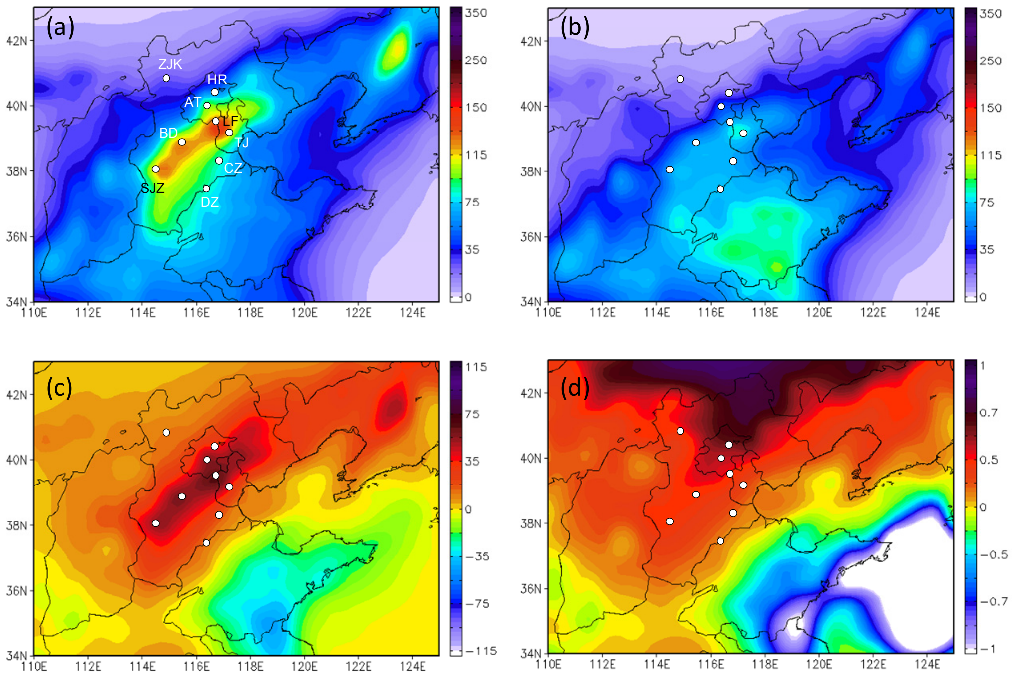

Figure S12. Spatial distributions of $PM_{2.5}$ (a) before APEC and (b) during APEC. (c) shows the difference before and during APEC, i.e., [Before APEC] – [APEC], and (d) shows the change ratio of $PM_{2.5}$ during APEC, which is ([Before APEC] – [APEC])/([Before APEC]). The maps were drawn by Igor Pro (version 6.3.7.2, WaveMetrics, Inc., Oregon USA), http://www.wavemetrics.com/.

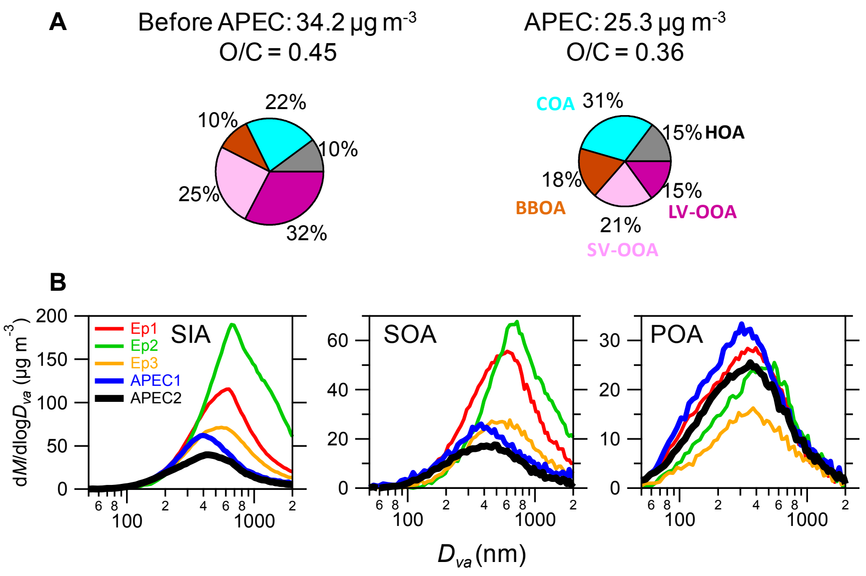

Figure S13. (A) Average organic aerosol composition and oxygen-to-carbon ratio (O/C) before and during APEC, (B) Average size distributions of SIA, SOA and POA during five episodes which are marked in Fig. 1.

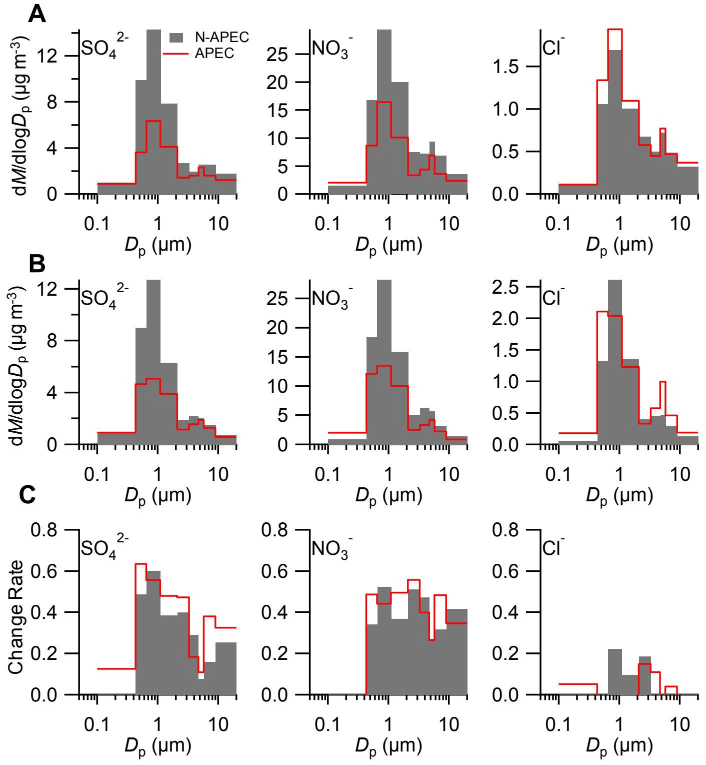

Figure S14. Size distributions of SO_4^{2-}, NO_3^-, and Cl^- from the size-segregated filter samples at (A) the ground site and (B) 260 m during N-APEC (10/30 17:00 – 11/3 10:00) and APEC (11/6 11:00 – 11/10 10:00). (C) shows the change rate of three species during the APEC which is calculated as [N-APEC – APEC]/[N-APEC].

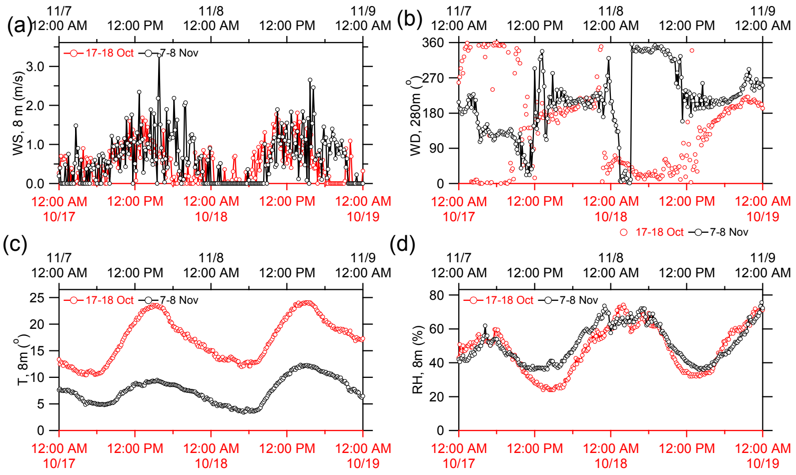

Figure S15. Variations of meteorological variables (a) wind speed, (b) wind direction, (c) temperature, and (d) relative humidity during two episodes, i.e., 17 – 18 October and 7 – 8 November. The two episodes show similar WS, WD, and RH with the largest difference in temperature.

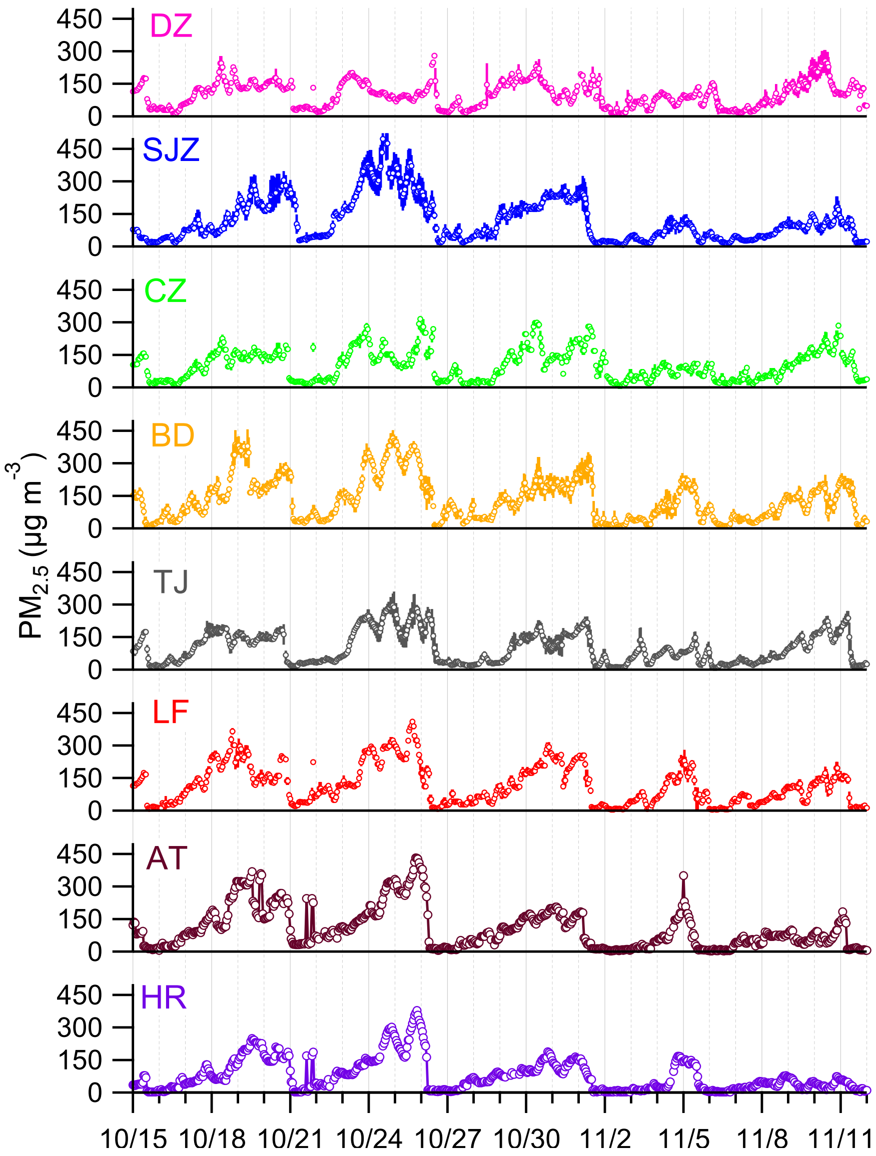

Figure S16. Time series of $PM_{2.5}$ mass concentration in different cities (see Fig. S11 for detail).

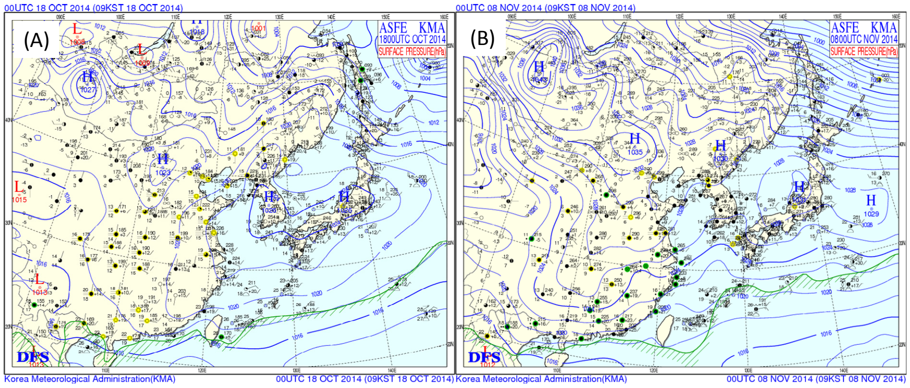

Figure S17. Weather analysis chart on (A) 18 October (8:00 am) and (B) 8 November (8:00 am). The charts were downloaded from http://web.kma.go.kr/eng/weather/images/analysischart.jsp.

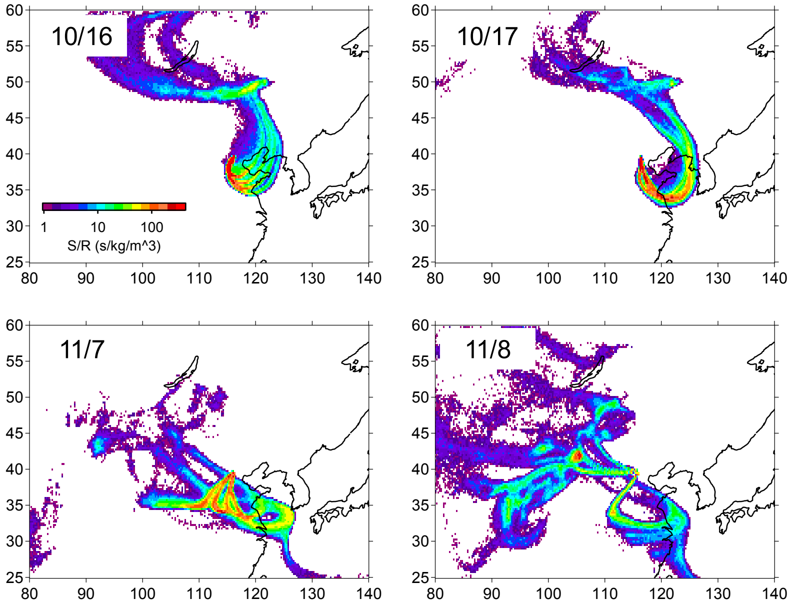

Figure S18. The footprint region (altitude below 100 m height in the planetary boundary layer (PBL)) of sampling at the site was determined by five days' backward simulations of air mass movement on the basis of the FLEXPART model, which is a Lagrangian particle dispersion model that has been widely applied in calculating long-range and meso-scale dispersion of air pollutants. A detailed description and validations of this model can be found in the literature [18]. In the present study, the FLEXPART model (Version 8.23) was driven by meteorological field (spatial resolution = 28 km, time resolution = 3 hours) from Global Forecast System produced by the National Centers for Environmental Prediction (NCEP). In simulations, 10,000 tracer particles were released from the site at a height between 200 and 500 m and the model was run backwardly to determine the source-receptor-relationship for specified observation period. The longer the tracer stayed in that cell, the greater the impact

from surface emission sources. The maps were drawn by Igor Pro (version 6.3.7.2, WaveMetrics, Inc., Oregon USA), http://www.wavemetrics.com/.

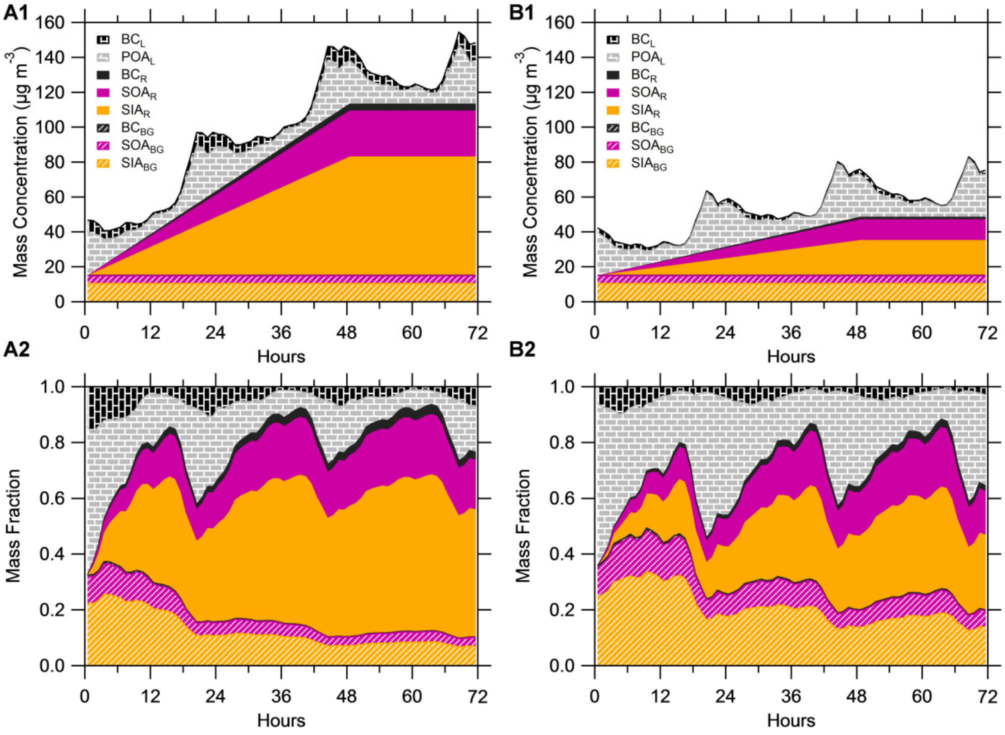

Figure S19. A conceptual framework to describe the 72 hr evolution of primary and secondary aerosol without emission control before the APEC (A1 and A2) and with the emission control during the APEC (B1 and B2). This figure is similar to Fig. 4 yet with the evolution of primary species starting from mid-night.

References:

1 DeCarlo, P. F. *et al.* Field-Deployable, High-Resolution, Time-of-Flight Aerosol Mass Spectrometer. *Anal. Chem.* **78**, 8281-8289 (2006).

2 Jimenez, J. L. *et al.* Ambient aerosol sampling with an Aerosol Mass Spectrometer. *J. Geophys. Res.-Atmos.* **108**, 8425, doi:doi:10:1029/2001JD001213 (2003).

3 Jayne, J. T. *et al.* Development of an aerosol mass spectrometer for size and composition analysis of submicron particles. *Aerosol Sci. Tech.* **33**, 49-70 (2000).

4 Sun, Y. L. *et al.* Size-resolved aerosol chemistry on Whistler Mountain, Canada with a high-resolution aerosol mass spectrometer during INTEX-B. *Atmos. Chem. Phys.* **9**, 3095-3111 (2009).

5 Kebabian, P. L., Wood, E. C., Herndon, S. C. & Freedman, A. A Practical Alternative to Chemiluminescence-Based Detection of Nitrogen Dioxide:

Cavity Attenuated Phase Shift Spectroscopy. *Environ. Sci. Technol.* **42**, 6040-6045, doi:10.1021/es703204j (2008).
6 Ge, B. *et al.* Nitrogen dioxide measurement by cavity attenuated phase shift spectroscopy (CAPS) and implications in ozone production efficiency and nitrate formation in Beijing, China. *Journal of Geophysical Research: Atmospheres* **118**, 9499–9509, doi:10.1002/jgrd.50757 (2013).
7 Yang, T. *et al.* Evaluation of the effect of air pollution control during the Beijing 2008 Olympic Games using Lidar data. *Chin. Sci. Bull.* **55**, 1311-1316, doi:10.1007/s11434-010-0081-y (2010).
8 Fernald, F. G. Analysis of atmospheric lidar observations- Some comments. *Appl Optics* **23**, 652-653 (1984).
9 Ruchith, R. D., Kalapureddy, M. C. R., Deshpande, S., Dani, K. K. & Ernest Raj, P. Inter-comparison of wind profiles in the tropical boundary layer remotely sensed from GPS radiosonde and Doppler wind lidar. *International Journal of Remote Sensing* **35**, 3300-3315, doi:10.1080/01431161.2014.902552 (2014).
10 Ng, N. L. *et al.* An Aerosol Chemical Speciation Monitor (ACSM) for routine monitoring of the composition and mass concentrations of ambient aerosol. *Aerosol Sci. Tech.* **45**, 770 - 784 (2011).
11 Sun, Y. L. *et al.* Characterization of summer organic and inorganic aerosols in Beijing, China with an Aerosol Chemical Speciation Monitor. *Atmos. Environ.* **51**, 250-259, doi:10.1016/j.atmosenv.2012.01.013 (2012).
12 Paatero, P. & Tapper, U. Positive matrix factorization: A non-negative factor model with optimal utilization of error estimates of data values. *Environmetrics* **5**, 111-126 (1994).
13 Ulbrich, I. M., Canagaratna, M. R., Zhang, Q., Worsnop, D. R. & Jimenez, J. L. Interpretation of organic components from Positive Matrix Factorization of aerosol mass spectrometric data. *Atmos. Chem. Phys.* **9**, 2891-2918 (2009).
14 Paatero, P. & Hopke, P. K. Discarding or downweighting high-noise variables in factor analytic models. *Anal. Chim. Acta* **490**, 277-289 (2003).
15 Zhang, Q. *et al.* Understanding atmospheric organic aerosols via factor analysis of aerosol mass spectrometry: a review. *Anal. Bioanal. Chem.* **401**, 3045-3067, doi:10.1007/s00216-011-5355-y (2011).
16 Zhang, Q., Worsnop, D. R., Canagaratna, M. R. & Jimenez, J. L. Hydrocarbon-like and oxygenated organic aerosols in Pittsburgh: Insights into sources and processes of organic aerosols. *Atmos. Chem. Phys.* **5**, 3289-3311 (2005).
17 Xu, J. *et al.* Chemical composition, sources, and processes of urban aerosols during summertime in northwest China: insights from high-resolution aerosol mass spectrometry. *Atmos. Chem. Phys.* **14**, 12593-12611, doi:10.5194/acp-14-12593-2014 (2014).
18 Stohl, A., Forster, C., Frank, A., Seibert, P. & Wotawa, G. Technical note: The Lagrangian particle dispersion model FLEXPART version 6.2. *Atmos. Chem. Phys.* **5**, 2461-2474 (2005).
